# A Blind Spot in Post‐Liver Transplant Care: Correlation and Causation in Immunosuppression, Drug Monitoring, and Immune Checkpoint Inhibitor‐Related Rejection

**DOI:** 10.1002/hcs2.70070

**Published:** 2026-04-16

**Authors:** Björn Nashan

**Affiliations:** ^1^ Research Centre of Big Data and Artificial Intelligence of Medicine, The First Affiliated Hospital, Sun Yat‐sen University Guangzhou China; ^2^ The Transplantation Center, First Affiliated Hospital, School of Life Sciences and Medical Center, University of Sciences and Technology of China Hefei China

**Abbreviations**
AR: acute rejection
HCC: hepatocellular carcinoma
ICI: immune checkpoint inhibitor
IS: immunosuppression
LT: liver transplantation

Correlation describes a relationship between two variables, where a change in one is associated with a change in the other, but it does not prove a cause‐and‐effect relationship. Causation, by contrast, means that a change in one variable directly produces or influences a change in the other. The well‐known principle that “*correlation does not imply causation*” underscores that two events occurring together do not necessarily indicate that one causes the other—both could be influenced by a third, unobserved factor.

In recent years, immune checkpoint inhibitors (ICIs) targeting PD‐1, PD‐L1, or CTLA‐4 have become pivotal systemic therapies for advanced hepatocellular carcinoma (HCC) [1, 2, 3, 4, 5, 6, 7]. By improving tumor response rates, ICIs have made successful downstaging before liver transplantation (LT) increasingly feasible, thereby expanding transplant eligibility. However, pretransplant ICI exposure has been associated with higher rates of acute allograft rejection (AR)—often exceeding 20%—with episodes that are typically more severe, resistant to treatment, and linked to greater graft loss. Although factors such as ICI washout duration, patient age, and drug type have been examined, their predictive value remains limited, and consensus on optimal management is lacking.

Most available data—whether from single‐center reports, multicenter retrospective studies, or registry analyses—demonstrate a correlation between ICI use and the incidence of AR. Many of these studies then infer a causal relationship, attributing increased rejection risk to systemic immune activation induced by ICIs. Immune‐related adverse events, reflecting persistent systemic inflammation, have indeed been linked to improved oncologic outcomes but may also signify heightened immune reactivity that predisposes to graft rejection.

The central question, however, remains: Does correlation prove causation?

We recently published a retrospective study assessing the incidence of AR after LT in HCC patients previously treated with ICIs, all managed within a fully standardized perioperative pathway [8]. Contrary to reports suggesting a substantially increased rejection risk after ICI exposure (Table 1), no AR episodes were observed among 59 transplanted patients, including 13 with relatively short ICI washout intervals.

**Table 1:** Studies on ICIs application in HCC liver transplantation.

Author and year	Study design	No. of patients	Control group	Primary treatments	Liver biopsy	Median TLAT (days)	Complications Clavien‐Dindo	Acute rejection rate (%)	Immuno‐suppressive regimen	Therapeutic drug monitoring
Wang et al. 2023[1]	Retrospective cohort study	16	No	Nivolumab (2); Pembrolizumab (7); Sintilimab (4); Camrelizumab (2); Nivolumab, Toripalimab, Sintilimab, and Tislelizumab (1)	Biopsy proven AR in 4 patients according to the Banff working group	21	—	56.3	Yes	Yes
Tabrizian et al. 2025[3]	Prospective multicenter	43	No	Nivolumab (68); Atezolizumab + Bevacizumab (24); Pembrolizumab (21); Durvalumab + Tremelimumab (4); 94% combined with locoregional therapy	Biopsy‐proven allograft rejection was graded histologically according to the classification proposed by the Banff working group.	43	—	16.3	No	No
Tabrizian et al. 2025[5]	Prospective multicenter	17	No	Atezolizumab + Bevacizumab (16 with locoregional therapy)	Yes, but no details	78	—	11.8	Yes	No
Aceituno et al. 2025[7]	Retrospective multicenter	152	No	Nivolumab (34); Atezolizumab + Bevacizumab (8); other ICIs (3)	No	58	—	28.3	No	No
Guo et al. 2024[6]	Retrospective multicenter	83	No	PD‐1 inhibitors (95.2%), PD‐L1 inhibitors (4.8%, Atezolizumab); combined with targeted/local therapy or resection	Overall, a total of 23 (27.7%) recipients developed post‐LT allograft rejection across 7 centers, including 7 (30.4%) diagnosed by liver biopsy and 16 (69.6%) diagnosed by clinical signs and hematological tests.	58	—	27.7	No	No
Lu et al. 2024[4]	Retrospective multicenter	39	No	Tislelizumab (11); Sintilimab (10), Camrelizumab (7); other ICIs combined with systemic/local therapy (11)	Liver biopsy is not mandatory for AR diagnosis, while if a biopsy is performed, the rejection activity index is defined according to the Banff score	50	—	23.1 (vs. 5 in non‐ICI group)	Yes	No
Fang et al. 2025[2]	Retrospective multicenter	209	No	Immunotherapy was primarily based on PD‐1 inhibitors (88.7%), with Camrelizumab (23.9%) and Sintilimab (23.9%) being the most used agents.	No data available	66	—	17.1	No	No
Wang et al. 2026[8]	Retrospective single center	59	Yes	Group 1 (*n* = 40): Transarterial chemoembolization (TACE), radiofrequency ablation (RFA), or resection Group 2 (*n* = 6): Targeted therapy alone Group 3 (*n* = 13) Camrelizumab (4); Tislelizumab (3); Sintilimab (3); Triprolizumab (1); Pembrolizumab (2)	No clinical indication	38	Group 1: *n* = 22 (55.0%) Grade I–II *n* = 15 (37.5%) Grade III *n* = 3 (7.5%) Grade IV Group 2: *n* = 4 (66.7%) Grade I–II *n* = 2 (33.3%) Grade III Group 3: *n* = 7 (53.8%) Grade I–II, *n* = 4 (30.8%) Grade III *n* = 2 (15.4%) Grade IV	0	Yes	Yes

Abbreviations: AR, acute rejection; HCC, hepatocellular carcinoma; ICI, immune checkpoint inhibitor; LT, liver transplantation; RFA, radiofrequency ablation; TACE, transarterial chemoembolization.

All patients were treated within a structured enhanced recovery after surgery‐based clinical pathway incorporating immunosuppressive induction, early mTOR inhibitor conversion, calcineurin inhibitor minimization, and rigorous therapeutic drug monitoring [9]. Clinical outcomes, complication rates, and immunosuppressive drug exposure were comparable between ICI‐exposed and non‐ICI‐exposed patients. Although 1‐year overall and recurrence‐free survival were lower in the ICI group, these differences may reflect higher baseline tumor burden rather than immunologic complications or rejection.

These findings challenge the assumption that prior ICI exposure per se drives post‐transplant AR. Instead, they suggest that rejection risk is strongly influenced by perioperative immunosuppressive strategy, standardization of care pathways, and the quality of immunosuppressive drug monitoring and reporting. When evaluating LT outcomes after ICI therapy, emphasis should shift from isolated immunologic attribution to protocol‐driven perioperative management.

In summary, here we have the first key conundrum: the tendency to attribute post‐liver transplant acute rejection to prior ICI exposure rather than to the adequacy of the immunosuppressive regimen and the rigor of therapeutic drug monitoring within standardized perioperative care pathways.

A second key conundrum, in addition to deficiencies in therapeutic drug monitoring, is the persistent tendency to equate elevated liver function tests with acute rejection, often without adequate diagnostic confirmation.

Diagnosis of AR is based on four main parameters:

1. Elevation of liver enzymes and/or cholestatic markers;
2. Impaired liver function reflected by altered coagulation parameters;
3. Histologic confirmation by biopsy;
4. Correlation with other parameters given by the differential diagnosis.

However, as straightforward as this may appear, the differential diagnosis is complex [10]. Key alternative explanations for impaired liver function in the early post‐transplant period include:

1. Inadequate immunosuppression (IS) exposure (e.g., subtherapeutic tacrolimus or sirolimus levels);
2. IS toxicity, particularly in the presence of CYP450 inhibitors such as antifungal agents;
3. Surgical complications (e.g., portal vein or bile duct stenosis, compressive hematoma);
4. Ischemia‐reperfusion injury, especially in marginal or donation after circulatory death grafts;
5. Viral infections, including cytomegalovirus, hepatitis B virus, or hepatitis C virus reactivation;
6. Limited biopsy sensitivity, as chimerism, ischemia‐reperfusion injury and viral infections may mimic AR histologically.

Accurate diagnosis therefore requires the integration of multiple parameters, including event timing, IS exposure levels, concurrent medications, donor organ quality, and the exclusion of surgical or infectious causes. Any potential event should be discussed during daily rounds, based on comprehensive chart reviews and thorough documentation.

The proposal to address this challenge through routine protocol biopsies is appealing; however, it remains questionable whether the inflammatory patterns defined in the Banff classification can reliably distinguish the underlying cause of inflammation. Histologic changes may reflect ischemia‐reperfusion injury, viral infection, acute rejection, or chimerism‐related immune activity consistent with subclinical graft‐versus‐host disease.

This brings us to a third key conundrum—the presumption that inflammatory changes within the graft always represent acute rejection and biopsy being the gold standard to diagnose.

The Banff Working Group classification [11] for acute rejection in LT is not designed to account for chimerism‐related immune phenomena or graft‐versus‐host disease [12, 13, 14]. It focuses on histopathologic features of alloimmune‐mediated rejection, such as portal inflammation, bile duct injury, and venous endothelitis, without distinguishing whether inflammatory infiltrates originate from recipient alloimmunity, donor‐derived lymphocytes, ischemia‐reperfusion injury, infection, or drug toxicity. Consequently, Banff‐defined inflammation may overlap with immune activity related to mixed chimerism or subclinical graft‐versus‐host disease. Histologic findings therefore require careful interpretation within a comprehensive clinical and immunologic context, as reliance on biopsy‐based criteria alone may lead to misclassification of inflammatory processes as acute rejection and inappropriate intensification of immunosuppression. Consequently, histopathological findings must be interpreted within a comprehensive clinical and immunological context, with particular caution, as inflammatory signals may not represent acute rejection but rather subclinical immune phenomena [12, 13, 14].

None of the currently published studies reporting a correlation between ICI exposure and AR provide this level of diagnostic rigor, instead an increase in liver function tests triggers immediately, as a self‐fulfilling prophecy, the diagnosis of AR sometimes supported with a liver biopsy. They also fail to present control groups, detailed IS protocols, therapeutic drug monitoring data, or standardized management pathways—essential elements for progressing from correlation to causation (Table 1). Robust control groups are indispensable to establish a true causal relationship between an intervention and its observed outcome.

To move beyond mere association and toward demonstrating causation, studies investigating the relationship between ICI exposure and acute rejection must be grounded in standardized perioperative management avoiding the aforementioned blind spots. Implementing clearly defined enhanced recovery after surgery protocols and structured clinical pathways—including transparent reporting of immunosuppressive regimens, therapeutic drug monitoring, and complication surveillance—is essential. Only through such uniform frameworks (including e.g., prospective data structures, prespecified confounder adjustment, or target‐trial‐like thinking where feasible) can data from different centers become comparable, confounding factors be minimized, and genuine causal mechanisms identified. Collaborative, prospective studies applying these standardized strategies are urgently needed to establish evidence‐based, reproducible conclusions regarding the true impact of ICI therapy on post‐transplant outcomes.

## Author Contributions

**Björn Nashan:** conceptualization, methodology, writing – review and editing, writing – original draft, validation.

## Funding

The author has nothing to report.

## Ethics Statement

The author has nothing to report.

## Consent

The author has nothing to report.

## Conflicts of Interest

Professor Björn Nashan is a member of the *Health Care Science* Editorial Board. To minimize bias, he was excluded from all editorial decision‐making related to the acceptance of this article for publication.

## Data Availability

Data sharing is not applicable to this article as no datasets were generated or analyzed during the current study.
